# Stat2 stability regulation: an intersection between immunity and carcinogenesis

**DOI:** 10.1038/s12276-020-00506-6

**Published:** 2020-09-25

**Authors:** Cheol-Jung Lee, Hyun-Jung An, Eun Suh Cho, Han Chang Kang, Joo Young Lee, Hye Suk Lee, Yong-Yeon Cho

**Affiliations:** 1 College of Pharmacy, The Catholic University of Korea, 43, Jibong-Ro, Wonmi-Gu, Bucheon-Si, Gyeonggi-Do, 14662 Republic of Korea; 2 College of Biological Sciences, University of Minnesota, 3-104 MCB, 420, Washington Ave, SE, Minneapolis, MN 55455 USA

**Keywords:** Melanoma, Protein-protein interaction networks

## Abstract

Signal transducer and activator of transcription (STAT2) is a member of the STAT family that plays an essential role in immune responses to extracellular and intracellular stimuli, including inflammatory reactions, invasion of foreign materials, and cancer initiation. Although the majority of STAT2 studies in the last few decades have focused on interferon (IFN)-α/β (IFNα/β) signaling pathway-mediated host defense against viral infections, recent studies have revealed that STAT2 also plays an important role in human cancer development. Notably, strategic research on STAT2 function has provided evidence that transient regulatory activity by homo- or heterodimerization induces its nuclear localization where it to forms a ternary IFN-stimulated gene factor 3 (ISGF3) complex, which is composed of STAT1 and/or STAT2 and IFN regulatory factor 9 (IEF9). The molecular mechanisms of ISGF3-mediated ISG gene expression provide the basic foundation for the regulation of STAT2 protein activity but not protein quality control. Recently, previously unknown molecular mechanisms of STAT2-mediated cell proliferation via STAT2 protein quality control were elucidated. In this review, we briefly summarize the role of STAT2 in immune responses and carcinogenesis with respect to the molecular mechanisms of STAT2 stability regulation via the proteasomal degradation pathway.

## Cancer: Significance of stability of regulatory protein

The activity of STAT2, a protein stimulated by molecular signalling systems to activate selected genes in ways that can lead to cancer, is regulated by factors controlling its rate of degradation. Yong-Yeon Cho and colleagues at The Catholic University of Korea in South Korea review the role of STAT2 in links between molecular signals of the immune response and the onset of cancer. They focus on the significance of factors that regulate the stability of STAT2. One key factor appears to be the molecular mechanisms controlling the degradation of STAT2 by cellular structures called proteasomes. These structures break down proteins as part of routine cell maintenance. Deeper understanding of the stimulation, action and degradation of STAT2 will assist efforts to treat the many cancers in which STAT2 activity is involved.

## Introduction

**Fig. 1:**
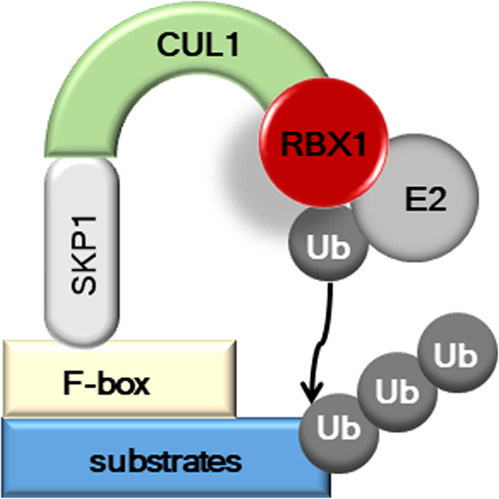
Basic molecular structure of the SCF complex. The SCF (SKP1-CUL1-F-box protein) ubiquitin ligase complex is the most well-characterized cullin RING ubiquitin ligase (CRL). Since the F-box proteins contain approximately 69 different components that bind to SKP1, many different combination of specific SCF complex formation are possible with a selective substrate and depending on the cellular context. Moreover, eight cullin proteins add a more complicated combinational probability for tissue, developmental, and stimuli specificities. Finally, the ubiquitination in mono-, multi-, single-chain, poly-chain, and branched-chain patterns might indicate precise regulation of the cellular functions of proteins by reflecting diverse cell conditions.

Our recent studies have demonstrated that signal transducer and activator of transcription 2 (STAT2) stability regulation plays an important role in melanoma formation. Serine/threonine phosphorylation of STAT2 by glycogen synthase kinase 3 α/β (GSK3α/β) enhances the recruitment of the S-phase kinase-associated protein 1 (SKP1)-cullin 1 (CUL1)-F-box protein-FBXW7 (SCF^FBXW7^) complex, resulting in STAT2 destabilization by ubiquitination-mediated degradation. There are several phosphorylation sites on the STAT2 protein. The ultimate fate of STAT2 protein, such as dimerization-mediated nuclear localization and activation, enhancement of transactivation activity, or destabilization, is dependent on its phosphorylation status at specific amino acid residues and the cellular context. Although the phosphorylation of a STAT protein is a predominant mechanism for regulating its activity, accumulating evidence indicates that regulation of the STAT protein level modulates STAT activity by ubiquitination and destabilization via the proteasomal degradation pathway^1^. The stability regulation of STAT proteins has been analyzed, and the recently published results focused on STAT1 and STAT3. The specific ubiquitin ligase for STAT1 and STAT4, known as STAT-interacting LIM protein (SLIM), was previously discovered^2^. SLIM was shown to contain the PSD95/Dlg/ZO-1 and LIM domains. The LIM domain, which forms a zinc-finger structure related to the really interesting new gene finger and plant homeodomain structures, exhibits E3 ligase activity. Thus, SLIM promotes both the ubiquitination and degradation of STAT1 and STAT4^2^. Moreover, *SLIM*-knockout mice show enhanced STAT1 and STAT4 protein levels, resulting in increased interferon (INF)-γ (IFNγ) production in type 1T helper (Th1) cells. These findings show SLIM to be the first known cellular ubiquitin ligase with specificity for STAT proteins^2^. Since INF imposes restrictions against viral infection, virus-mediated destabilization of STAT proteins, which involves hijacking cellular factors that target STAT proteins, is an important molecular mechanism for viral evasion. A shorter protein half-life of STAT1 was shown to be triggered by infection with paramyxoviruses^3^, mumps virus^4^, and Sendai virus^5^. The ubiquitination and proteasomal degradation of STAT2 was shown to be caused by stimulation with paramyxoviruses^3^, cytomegalovirus^6^, and dengue virus^7^. These viral infections seem to recruit STAT1/2 proteins to the CUL4A-based ubiquitination complex for the proteasomal degradation of STAT1/2^8^. The data indicate that STAT protein activity can be modulated through protein stability regulation. The biological processes of the ubiquitin-proteasome system are mediated by three enzymatic reactions involving ubiquitin-activating E1 enzyme, ubiquitin-conjugating E2 enzyme, and ubiquitin–protein E3 enzyme. Thus, E3 ligases play a crucial role in determining target proteins for ubiquitination and degradation by E1–E3 enzymes^9^. Functional analysis of the F-box protein has demonstrated that the C-terminal domain (CTD) and F-box domain (FD) contribute to the selection of specific substrates for degradation. The CTD is involved in binding to specific substrates, while the FD acts as a protein–protein interaction domain by directly binding with adaptor protein SKP1 and recruiting F-box protein to the SCF complex^10^ (Fig. 1).

Since STAT2 plays an important role in host defense against viral infection and carcinogenesis, we provide a brief review of STAT2 activity regulation and discuss the novel role of STAT2 activity regulation in carcinogenesis, especially melanoma.

### STAT2 among STAT proteins in carcinogenesis

The Janus kinase (JAK)–STAT pathway plays a crucial role in signal transduction and cellular responses to various cytokines. Immediately after the first direct linkage between STAT protein and carcinoma in humans was discovered, based on the constitutive activation of STAT3 playing a key role in the carcinogenesis of head and neck cancer and in multiple myeloma cells^11,12^, research on the involvement of STAT proteins in human cancers has been logarithmically expanded in recent decades. Subsequent research has revealed that several types of solid tumors, including leukemia and lymphomas, are linked with the constitutive activation of STAT3. Interleukin (IL)-6 (IL-6) autocrine or paracrine loops have been recognized inducers of constitutive STAT3 activity in myeloma and prostate malignant cell lines^13^. Moreover, the role of STAT proteins is not limited to cytokines. For example, transforming growth factor-α (TGF-α)-mediated epidermal growth factor receptor (EGFR) signaling was shown to play a vital role in the activation of STAT3 in certain head and neck cancer cell lines^12^, and growth factor-mediated receptor tyrosine kinase signaling pathways such as that of the hepatocyte growth factor signaling via receptor c-MET were shown to be related to the transformation of leiomyosarcoma cells, breast carcinoma cells, melanoma cells, and lung cancer cells in conjunction with SRC kinase, which stimulates the expression of STAT3^14–17^. On the other hand, evidence indicating that the antagonization of STAT3 signaling induces cell death in human U266 myeloma cells^11^ provides a good reason to develop STAT inhibitors as key tools to treat human cancers. Although STAT proteins play an essential role in cancer, research in the fields of immunology and oncology has mainly focused on STAT1/3.

STAT2, another member of the STAT family, is considered a hallmark of INF-I (IFNα/β) IFN-III (IFNλ1)/IL-29, IFNλ2/IL-28A, and IFNλ3/IL-28B activation^18^. The responsiveness of immune and nonimmune cells to INF-I and -III is known as the classic trigger for the formation of a heterotrimer complex known as ISGF3, comprising STAT2, STAT1, and interferon regulatory factor 9 (IRF9). Since STAT2 is distinct from other members of the STAT family in that it does not form a homodimer to recognize a DNA target, the formation of the heterotrimeric ISGF3 complex is a unique characteristic among STAT-dependent pathways. However, alternative STAT complexes composed of homo- and heterodimers of other STAT family members are known to form upon IFN-I stimulation in a cell-specific fashion^19,20^. Since keratinocytes are not only a mechanical barrier to outside invaders but also immunocompetent cells that can produce various inflammatory cytokines, ultraviolet irradiation has been used to shown a dramatically increased production of IL-1, -3, and -6, tumor necrosis factor, and granulocyte/macrophage-colony stimulating factor by these epidermal cells^21^. Our research group reported that STAT2 protein levels are higher in melanoma tissues than in normal skin tissues. Melanoma cell proliferation is also correlated with STAT2 protein levels, indicating that STAT2 activity plays an essential role in melanoma promotion. In summary, the important finding is that STAT2 stability regulation is directly involved in melanoma cell proliferation^22^. Moreover, the interaction of STAT2 with FBXW7 is stimulated by ultraviolet B (UVB) irradiation, resulting in a reduction in STAT2 protein. Notably, genetic deprivation induced by sh-RNA STAT2 suppressed SK-MEL-28 cell proliferation, whereas ectopic overexpression of STAT2 enhanced SK-MEL-2 cell proliferation. These results also support our conclusion that STAT2 is a key player in the regulation of melanoma promotion.

### Functional anatomy of STAT2

#### Roles of STAT2 domains

The STAT family consists of seven transcription factors, each of which contains seven structurally and functionally conserved domains: N-terminal domain (NTD), coiled-coil domain (CCD), DNA-binding domain (DBD), linker domain (LD), Src-homology 2 domain (SH2D), tyrosine-phosphorylation site (pY), and transcriptional activation domain (TAD)^23^. Among these domains several regions have highly homologous amino acid sequences, including SH2D, which is involved in the activation and dimerization of STAT proteins^24,25^, DBD^26^, and a transactivation domain located in the carboxyl terminus^27,28^.

In terms of amino acid alignment, STAT2 is the longest and largest isotype among human STAT family members, with a molecular weight of 113 kDa consisting of 851 amino acid residues, whereas mouse STAT2 is even longer and larger, consisting of 923 amino acid residues and with a molecular weight of 130 kDa^29^. Despite the structural differences between humans and mouse STAT2, responsiveness to IFNα stimulation was fully restored in human STAT2-knockout cells by the introduction of mouse STAT2^30^. This functional compensation provided by mouse STAT2 is also corroborated by a report indicating that the overexpression of human STAT2 in STAT2-deficient mouse fibroblasts was shown to complement defects in IFN-I signaling^31^. These results indicate that the role of STAT2 in physiological conditions is functionally unique and well conserved.

**Fig. 2:**
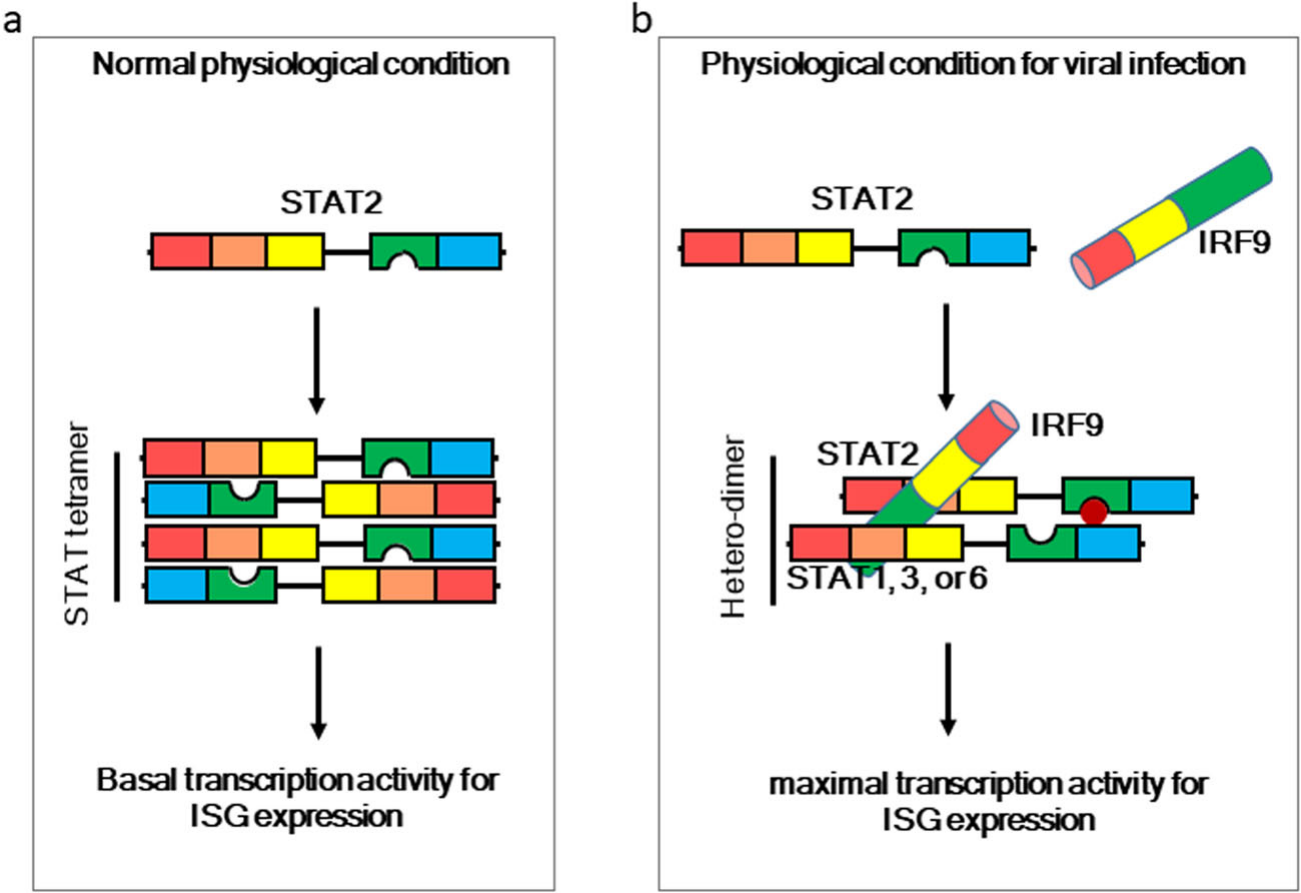
Representative regulatory mechanisms of STAT2-mediated ISG expression. **a** ISG expression under normal physiological conditions. Without stimulation, STAT2 forms a tetramer complex with other STAT family members, including STAT1, 3, and 6, through their N-terminal domains, resulting in an anti-parallel structure. The complex induces basal expression of target genes. **b** ISG expression under physiological conditions after viral infection. When cells are infected with viruses, activated JAK1- and TYK2-mediated phosphorylation of STAT2 at tyrosine residues triggers the formation of a SH2–pTyr interaction-mediated homo- or heterodimer complex, which associates with IRF9 and results in the formation of the ISGF3 complex. The ISGF3 complex plays a key role in target gene expression in response to viral infection.

Scientists initially believed that STAT proteins were monomers prior to their activation by tyrosine phosphorylation. However, accumulating structural and functional evidence indicates that multiple unphosphorylated STAT proteins (U-STATs) already exist as dimers in living cells. The NTD (1–130 amino acid residues) of STAT2 consists of eight short α-helices and is involved in the formation of U-STAT dimers with anti-parallel conformations (Fig. 2)^32^. As SH2Ds of STAT proteins have been shown to interact upon tyrosine phosphorylation^33^, it is generally well accepted that tyrosine phosphorylation of SH2D is indispensable for homo- or heterodimerization, nuclear localization and DNA binding. Importantly, STAT1/2 proteins lacking tyrosine phosphorylation are still able to combine with IRF9 to form unphosphorylated ISGF3 (U-ISGF3), which sustains the transcription of approximately one-quarter of the initially induced ISGs during the late phase response to IFN-I^34^. Moreover, STAT activity is generally regulated at the N-terminal region by the formation of STAT tetramers^35^ and tyrosine dephosphorylation^36^. Thus, the literature strongly suggests that the NTD of STAT2 plays a role in the basal transcriptional expression of ISG.

**Fig. 3:**
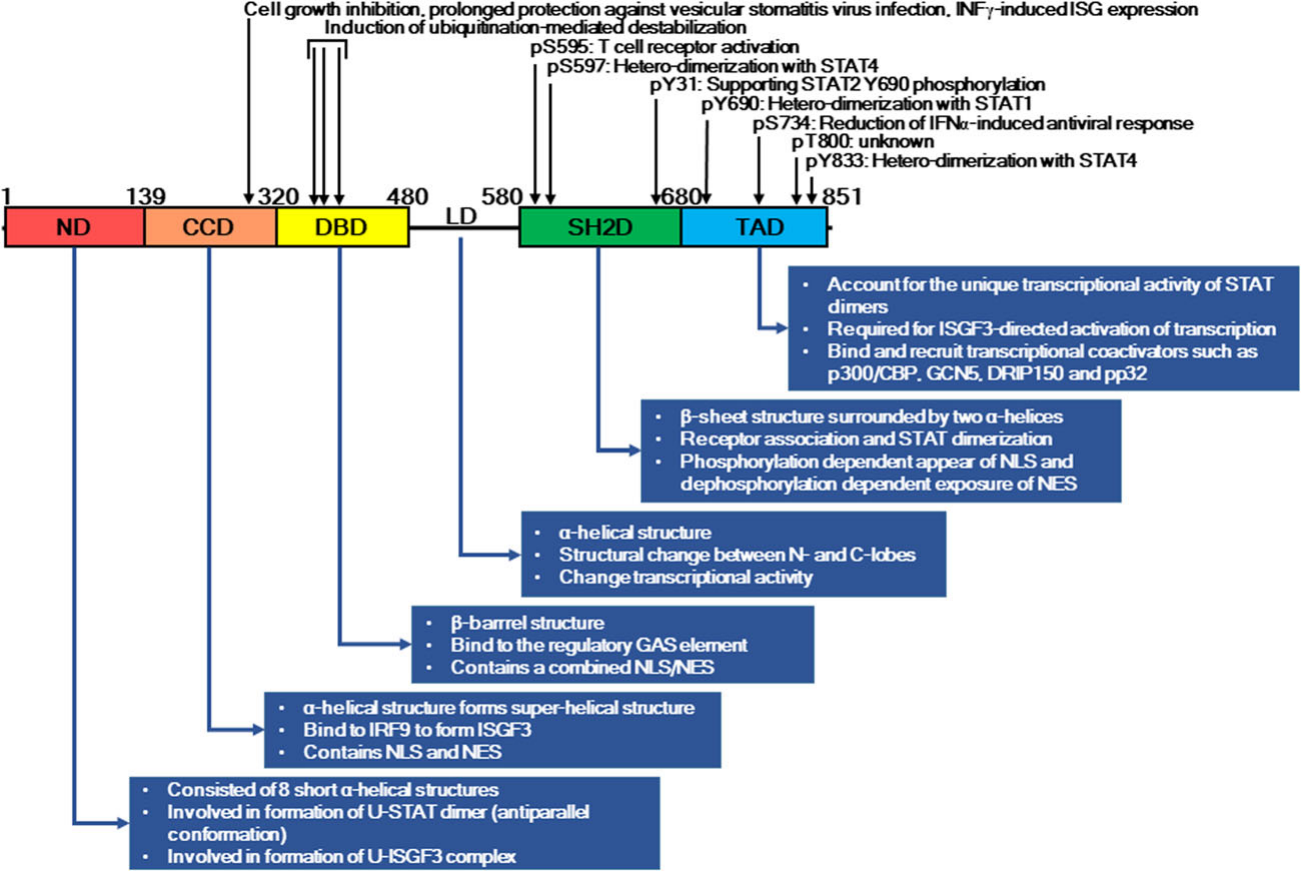
Characteristics and roles of STAT2 domains. Each STAT2 domain plays a specific role in homo- or heterodimerization and ISG expression. The diverse stimuli from outside or inside cells induce posttranslational modifications such as phosphorylation and ubiquitination to induce structural changes, parallel or anti-parallel oriented homo- or heterodimers, nuclear or cytosolic localization, and DNA binding.

The CCD spanning amino acids (aa) 135–315 in STAT2 has a superhelical structure consisting of four α-helices wrapped around each other. The CCD mediates protein–protein interactions with IRF9, which is a member of the IRF family. The IRF family generally shares amino acid homology in the N-terminal DBD, which is characterized by a tryptophan repeat^37^. Moreover, a bipartite basic nuclear localization signal (NLS) in the DBD of IRF9 is recognized by importin-α adapter family proteins, including importins-α3, -α5, and -α7^38^. CRM1/exportin 1-dependent nuclear export of various STAT proteins has been shown to be mediated through the CCD^39^. Thus, the CCD of STAT2 in conjunction with the carboxyl terminus of IRF9 plays a key role in the formation of the ISGF3 complex, resulting in the regulation of nuclear import and export by the formation of the importin-α:importin β1 complex^38^. Based on these data, the CCD of STAT2 plays a key role in ISGF3 complex formation and subcellular distribution by protein–protein interactions (Fig. 3).

The STAT protein family consists of transcription factors that regulate target gene expression by recognizing and binding to promoter regions. The β-barrel structure of the DBD spanning aa 320–480 is a highly conserved structure among all STAT family members and is critical for binding to the regulatory γ-activated sequence (GAS) element in STAT-target genes. Since the STAT2 homodimer is unable to bind to GAS sites, alternative STAT2-containing ISRE- or GAS-binding complexes involved in IFN-I signaling are composed of STAT2-containing heterodimers, namely, STAT1/2^40^, STAT2/3^41^, and STAT2/6^42^. The DBD domain of STAT2 contains a combined NLS/NES (nuclear export signal) that becomes active only upon phosphorylation-induced dimer formation^43,44^. Both CCD and DBD are also implicated in the interaction between U-STATs^45^. This collective evidence suggests that STAT proteins have functional diversity and are regulated by diverse intrinsic and extrinsic stimuli (Fig. 3).

The LD spanning aa 480–580 harbors mostly an α-helical structure and distinguishes the DBD from the SH2D that follows it. The relationship of LD with DBD and SH2D was recently investigated in terms of structural change, and the transcriptional activity of STAT proteins was found to be altered^46^. Recent studies have indicated that mutations of highly conserved residues in the LD of STAT1, such as K544A/E545A, fail to stimulate transcription because they release DNA more quickly than the tyrosine-phosphorylated dimer^47,48^. Based on these results, communication between the DBD and LD, as well as between the LD and SH2D, affects STAT functions such as DNA binding and transcriptional activation^46^. However, no evidence has been provided regarding the role of the LD in STAT2 (Fig. 3).

The STAT2 SH2D spanning aa 580–680 consists of a β-sheet surrounded by two α-helices and participates in STAT dimerization and receptor association by binding to phosphorylated tyrosine residues at a conserved arginine residue. The tyrosine residue of STAT2, Tyr690, located directly adjacent to SH2D plays an essential role in domain interactions with other STAT proteins, especially STAT1^27^. IFN-II induces the activation of STAT1 through phosphorylation of Y701 by IFN receptor-associated JAK1 and JAK2, leading to dimerization and nuclear accumulation of STAT1^49^. To interact with importin-5α^50^, the specific surface area produced by the newly formed and activated STAT1 dimer includes regions of the NTD^51^ and DBD^52^. Since tyrosine-phosphorylated STAT1 homodimers and STAT1–STAT2 heterodimers are rapidly imported to the nucleus, nuclear export is important in regulating the cellular localization of signaling proteins such as STAT proteins^38^. The DBD of STAT1 contains an NES that can function autonomously and appears to be masked when STAT1 dimers are bound to DNA^38^. Dephosphorylation in the nucleus releases STAT1 from DNA and exposes the NES to CRM1, resulting in the redistribution of STAT1 back to the cytoplasm^38^. Moreover, STAT2 is critical to the nuclear export of STAT2–IRF9 and ISGF3. Although STAT2 has been observed in the nucleus, we confirmed that STAT2 is predominantly located in the cytoplasm^22^. The other domain contained in IRF9 essential for interplay with the CCD of STAT2 is the IRF-association domain (Fig. 3).

The transcriptional activation domain (TAD), accounting for the unique transcriptional activity of STAT dimers upon binding to DNA, is located at the C-terminal end^53,54^. STAT2 together with STAT1 and IRF9 forms the ISGF3 complex in which TAD of STAT2 contributes without directly contacting DNA, whereas STAT1 stabilizes the complex by providing additional DNA contacts^55^. STAT2–TAD is required for ISGF3-directed activation of transcription^56,57^ and has been shown to bind and recruit transcriptional coactivators such as p300/CBP, GCN5, DRIP150, and pp32^28,56^. Moreover, a dominant NES in the C-terminus of STAT2 was shown to play a critical role in returning the STAT2–IRF9 complex back to the cytoplasm^38^. Although STAT2/IRF9 continually shuttles in and out of the nucleus, the U-STAT2 NES leads to its prominence in the cytoplasm (Fig. 3). Thus, the cytoplasmic localization of STAT2 dramatically changes following IFN-I and -III stimulation.

#### General activation mechanism of STAT2

IFNs are comprised of three main subfamilies designated type I, type II, and type III. Type I interferon (IFN-I) consists of IFNβ, IFNκ, IFNω, IFNε, and 13 subtypes of IFNα; type II interferon (IFN-II) consists of single IFNγ; and type III interferon (IFN-III) comprises IFNλ1, IFNλ2, IFNλ3^32,58^, and IFNλ4^25^. IFNs are mainly produced by plasmacytoid dendritic cells in response to stimulation of pattern recognition receptors, which are located on the cell surface, in the cytosol, and/or in endosomal compartments^59^, by microbial products or by foreign nucleic acids^60^.

The recognition and binding of IFNβ and all IFNα subtypes by a heterodimeric transmembrane receptor composed of IFNAR1 and IFNAR2 subunits initiate a signaling cascade mediated through the JAK–STAT pathway. Binding of INF-I to its cognitive IFN receptor that is preassociated with receptor-associated kinases, TYK2 and JAK1, results in activation by transphosphorylation^61^. This signaling pathway is a canonical cascade that transduces many cytokine and growth factor activation signals to the nucleus to activate transcription factors to enhance the expression of target genes. JAKs are defined by seven Janus homology (JH) domains, termed JH1–7. JH1 is a kinase domain that plays an important function in JAK enzymatic activity and the phosphorylation of STAT proteins, and JH3–JH4 of JAKs share homology with SH2Ds^29^. Although many publications initially reported that STAT proteins are monomers prior to their activation by tyrosine phosphorylation, accumulating evidence supported by structural and functional analyses indicates the existence of U-STATs as dimers in living cells^29^. These conclusions are based on findings that SH2Ds do not participate in this type of dimerization; both ends of the dimer structure are distinct, with STAT dimers adopting an anti-parallel conformation through the NTD^32,58^.

Tyrosine-phosphorylation results in the reciprocal binding of one phosphorylated STAT to another, forming either a homo- or heterodimer. INF-I-induced phosphorylation of STAT proteins at tyrosine residues leads to the dimerization of STAT proteins via a mutual SH2–pTyr interaction-mediated parallel dimer conformation^25,33^. However, in this case, the NTD is dispensable. In the canonical pathway of IFN-I-induced signaling, the phosphorylation of STAT1 at Tyr701 and STAT2 at Tyr690 induces heterodimerization in the parallel conformation and the interaction with IRF9 to form the ISGF3 complex^62^. On the other hand, the ISRE sequence is recognized by a cellular factor in response to IFNα treatment^63^. Subsequent studies have revealed that this cellular factor (ISGF3 complex) consists of four existing peptides, three of which have molecular weights of 84, 91, and 113 kDa and are currently known as STAT1β, STAT1α, and STAT2, respectively^64,65^. Dimer formation is the critical step in nuclear translocation since the NLS is formed by parts of the dimerized STAT DBDs^66^. The ISGF3 complex is translocated into the nucleus, whereupon the ISGF3-containing STAT dimer binds to gene promoters, specifically the IFN-I-stimulated response element (ISRE), which harbors the consensus sequence AGTTTCN2TTTCN, leading to the activation of transcription of over 300 interferon-stimulated genes (ISGs)^62^.

#### Tyrosine phosphorylation

Upon the binding of INF-I to IFNARs, activated TYK2- and JAK1-mediated transphosphorylation of INFARs in the intracellular region create docking sites for STAT1 and STAT2. Docked STAT1 and STAT2 are then phosphorylated by JAK1 and TYK2 at tyrosine residues, which can trigger the formation of homo- or heterodimers mediated by the SH2–pTyr interaction, especially the phosphorylation of STAT1 at Tyr701 and STAT2 at Tyr690. The association of the STAT1–STAT2 heterodimer with IRF9 results in the formation of the ISGF3 complex^62^, which plays a key role in target gene expression in response to viral infection^29^ and cancer cell growth. On the other hand, an important role for tyrosine phosphorylation was observed upon the partial deletion of the TAD, harboring aa 800–832, in STAT2, as deletion of this region was found to abolish the transcriptional activity of ISGF3^56^. Another study corroborates the possible phosphorylation of Thr800 and Tyr833 by STAT2-mediated ISGF3 activity^67–69^. Currently, although a mutation of Y833 to a phenylalanine residue was shown to impair the dimerization of STAT2–STAT4 induced by IFN-I^68,69^, the biological significance of Thr800 and Tyr833 in their phosphorylated states has not been elucidated.

#### Ser/Thr phosphorylation

In eukaryotes, serine and threonine phosphorylation is more common than tyrosine phosphorylation. Although tyrosine phosphorylation at the TAD of STAT proteins is a well-known event that plays an essential role in STAT localization and activity, serine phosphorylation also plays a key role in modulating the transcription factor function of STAT proteins. The mutation of Ser727 in STAT3 to alanine (STAT3–S727A) abrogates the DNA-binding ability of STAT3. S727–STAT1 phosphorylation is necessary for STAT1 homodimerization to induce a strong transcriptional response during IFN signaling^70^. Importantly, STAT1β, an alternative splicing product of STAT1, does not harbor Ser727 and is interchangeable with STAT1α for target gene expression. These results provide a key explanation for the role of STAT1 phosphorylation at Ser727, indicating that it is not involved in ISGF3-mediated ISG expression^28^. More recently, STAT2 phosphorylation at Ser595 was identified in a phosphoproteomic screening performed to identify the phosphorylation events in Jurkat cells responding to T-cell receptor activation^71,72^. Importantly, STAT2 Ser595 phosphorylation was not uniquely induced by T-cell receptor activation, indicating that other signaling pathways induce Ser595 phosphorylation, depending on the cellular context. Another phosphoproteomic study identified additional phosphorylation sites in STAT2, including Thr800 in human colorectal cancer tissues^69^. Computational prediction grafting revealed STAT2 phosphorylation sites at Thr597 and Tyr833, which are located in the C-terminus and are involved in heterodimerization with STAT4 by IFN-I stimulation^67^. The phosphorylation of another serine residue in STAT2 was identified by mass spectrometry, indicating that Ser287 phosphorylation of STAT2 is induced in response to IFNα treatment^73^. The mutation of STAT2-S287A enhances IFNα-mediated cell responsiveness, including cell growth inhibition, prolonged protection against vesicular stomatitis viral infection, and IFN-induced ISG expression^73^. Notably, the phosphomimetic mutation of STAT2-S287D results in opposite responsiveness, likely due to loss of function^73^. Three years later, the same research group found that STAT2 is phosphorylated at Ser734, resulting in a reduction in the IFNα-induced antiviral response^74^. The mutation of STAT2 at Ser734 was shown to enhance IFNα-driven antiviral responses compared to those driven by wild-type STAT2. Moreover, a small subset of IFN-I-stimulated genes was shown to be upregulated by IFNα to a greater degree in cells expressing S734A–STAT2 than in cells expressing wild-type STAT2^74^. More recently, phosphorylation was found in the DBD of STAT2. The phosphorylation sites Ser381, Thr385, and S393 of STAT2 were confirmed by an in vitro kinase assay using wild-type and alanine-mutated STAT2 proteins, [γ-^32^p]ATP, and active GSK3α/β^22^. Importantly, GSK3α and GSK3β were found to phosphorylate STAT2 equally, resulting in the destabilization of STAT2 by ubiquitination^22^. These results suggest that the phosphorylation status of STAT2 is differentially regulated depending on the cellular context. Moreover, the kinases that phosphorylate serine and/or threonine residues in STAT2 have not been clearly elucidated.

### Physiological roles of STAT2 in human cancer

Not all malignant cells manifest as fully fledged tumors, and not all tumors metastasize. The general consensus is that these outcomes are due to the activity of immune cells and immune-regulatory factors driven mainly by JAK–STAT signaling^75^. Nonspecific responses to tumor cells are thought to be mitigated by natural killer (NK) cells. Thus, the JAK–STAT pathway has been hypothesized to tightly regulate the development, maturation, activation, and function of NK cells^75^. Moreover, the cytotoxic and lytic functions of activated CD8^+^ T cells and NK cells are triggered and enhanced by IFNγ, indicating that INF-II plays a more direct role in tumor cell killing. However, the roles of interferons in cancer development remain controversial. In addition, since interleukins such as IL-1, IL-6, IL-8, IL-10, tumor necrosis factors, and IFNs secreted by tumor-infiltrating lymphocytes are able to stimulate tumor cell proliferation, protect tumor cells from apoptosis, or promote angiogenesis and metastasis, the molecular mechanisms of these diverse effects of cytokines on cancer development and chemoresistance still need to be elucidated. For example, lung metastasis of melanoma and peritoneal dissemination of ovarian cancer are facilitated by IFNγ^76,77^. Furthermore, IFNγ promotes the epithelial-to-mesenchymal transition by IFIT5-mediated microRNA processing, resulting in renal cancer invasion^78^. Moreover, significant upregulation of several key effectors, including IFNGR1, IFNGR2, STAT1, and STAT2, in the IFNγ signaling pathway has been observed in metastatic renal cell carcinoma^79^. IL-6 is known to act as a protumorigenic cytokine by facilitating cell growth and anti-apoptosis in multiple myeloma^80^. As described above, STAT2 plays a fundamental role in ISGF3; the STAT2 homodimer can induce IL-6 gene expression either directly or as a part of the ISGF3 complex^81^. Several studies have shown that during the tumor initiation process, IL-6 is required in response to activated EGFR and K-RAS signaling^82^. In a STAT2-knockout mouse experiment, STAT2 deficiency was shown to inhibit colorectal carcinogenesis because of lower levels of inflammatory cytokines^83,84^, and mice lacking STAT2 but not STAT1 or IFNARs were found to be hypersensitive to LPS^84^. Moreover, the STAT2-mediated NF-κB signaling pathway additively supports carcinogenesis and cancer therapeutic drug resistance. For example, cells expressing the transactivation-deficient triple mutant p53-D281G (L22Q/W23S) were shown to exhibit enhanced cell growth, survival, and adhesion. Although a p53 mutant did not bind to the NF-κB2 promoter, STAT2 and CBP were found to be enriched at this promoter in p53-mutant H1299 cells but were not enriched in cells expressing wild-type p53^85^. In addition, some p53-mutant phenotypes show increased NF-κB2 expression^86^. The molecular mechanisms driving STAT2 participation in inflammatory responses were recently elucidated, and it was shown that the cooperation of IRF6-unphopshorylated STAT2-mediated NF-κB signaling drives IL-6 expression^87^. In addition, both IL-1α and IL-1β aggravate tumor angiogenesis and invasiveness via the induction of vascular endothelial cell growth factor and tumor necrosis factor^88^. However, the role of the STAT2-mediated signaling pathway in carcinogenesis has not been clearly defined. A recent publication provided further understanding of the physiological relevance of STAT2 protein levels in human cancers, especially melanoma proliferation^22^. The discovery of FBXW7 as a STAT2 stability regulator has deepened our understanding of the role of FBXW7 in human cancers. Approximately, 6% of all primary human cancers contain FBXW7 mutations, indicating that FBXW7 plays a tumor suppressive role^89^. When only melanoma cases are observed, the mutation rate of FBXW7 is increased to 8.1%^90^. Our study demonstrated that FBXW7 protein levels were significantly lower in melanoma tissues than in normal skin tissues^22^. In contrast, STAT2 protein levels were significantly elevated in melanoma tissues compared to normal skin tissues in humans^22^. This phenomenon was also observed when tissue array samples were expanded to include 10 normal skin tissues and 70 skin cancer tissues, including squamous cell carcinoma, basal cell carcinoma, and melanoma. Importantly, a Kaplan–Meier analysis indicated that high levels of STAT2 protein may reduce the 5-year survival probability for melanoma patients^22^. Thus, the FBXW7–STAT2 metabolic axis might be a potential target for melanoma treatment.

### STAT2 ubiquitination

Among posttranslational modifications, protein ubiquitination plays a key role in regulating diverse intracellular signaling pathways and biological processes^91–93^, including the mediation of host protection and cell growth, proliferation, and viability. While signaling to STAT1 and STAT2 is initiated and regulated through the transient activity induced by IFN stimulation, the extent of IFN signaling is tightly regulated at many levels to limit detrimental effects. Notably, ubiquitination is important in modulating IFN signaling^94^. The IFNAR1 chain of the IFN-I receptor is a key signaling mediator. The ubiquitination and degradation of IFNAR1 is mediated by a cullin-based E3 ubiquitin ligase^95^ via interactions with substrate-recognizing F-box proteins, such as βTrcP^96^. βTrcP tethering to the IFNAR1-SCF^SKP1/Cullin1/Rbx1^ complex facilitates the polyubiquitination of IFNAR1 predominantly within a cluster of three lysine residues^97,98^. In brief, a highly conserved Pro470 residue of human IFNAR1 following the endocytic motif induces a turn that orients the polypeptide chain of IFNAR1 in a position parallel to the membrane. This conformational change is induced by ubiquitination, resulting in the exposure, enhanced recognition and subsequent binding by AP50, which is a clathrin adaptor protein. Based on these observations, both Lys48- and Lys63-linked polyubiquitin chains contribute significantly to IFNAR1 endocytosis and subsequent degradation^98^. Polyubiquitination-mediated internalized IFNAR1 might be degraded through the lysosomal degradation pathway^98^. In the absence of ubiquitination, this motif is masked by associated TYK2, thereby preventing basal endocytosis and degradation of IFNAR1^99,100^. A key event in regulating IFNAR1 ubiquitination is its phosphorylation at specific serine residues following IFN-I engagement. One important clue to phosphorylation-mediated ubiquitination was found to be the recruitment of βTrcP and the remaining components of the E3 ubiquitin ligase, resulting in the ubiquitination at Lys501, Lys525, and Lys526 located proximal to the destruction motif^97^. The consequent increase in IFNAR1 ubiquitination and destabilization limits any further IFN responses.

**Fig. 4:**
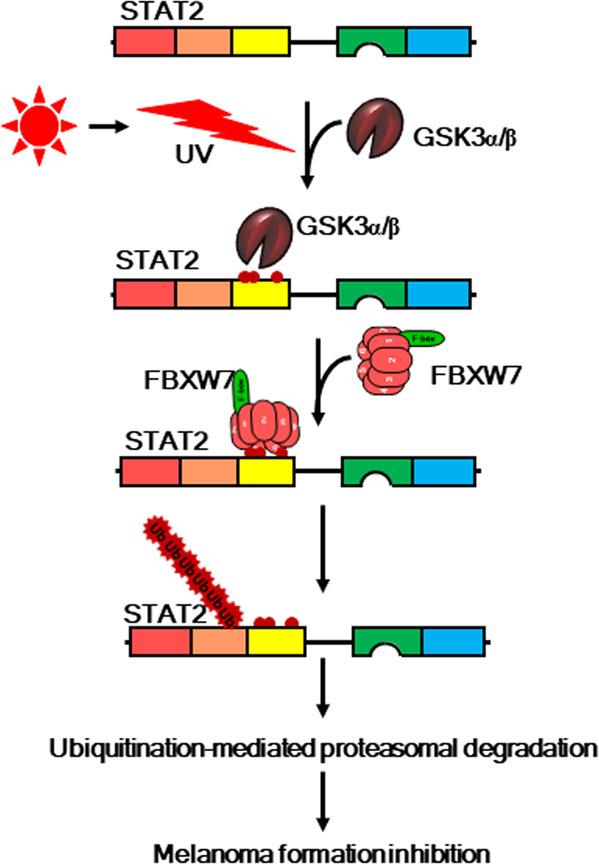
New paradigm for the regulation of STAT2 protein in melanoma formation. In normal cells, STAT2 protein levels are maintained at low levels. When cells are stimulated by UV, especially UVB, GSK3α/β phosphorylates STAT2 at the DBD, which recruits the SCF^FBXW7^ complex to form the SCF^FBXW7^–STAT2 complex. FBXW7 catalyzes K48 STAT2 polyubiquitination, resulting in a reduction in STAT2 protein levels via the proteasomal degradation pathway. Thus, cell proliferation at the acute stage is initially suppressed after UVB irradiation. However, in cancer cells with FBXW7 mutation(s), higher STAT2 protein levels are sustained compared to that of normal cells, resulting in the induction of cell proliferation.

The ubiquitination of STAT proteins was first reported by Kim et al.^101^, who found that IFN-γ-activated STAT1 regulates STAT1 protein levels via the ubiquitin-proteasome pathway. Although it was reported that βTrcP-based E3 ubiquitin ligase may also be involved in the ubiquitination and degradation of STAT1 phosphorylated by extracellular-signal-regulated kinase (ERK1/2) at Ser727^102^, the identities of the cellular E3 ubiquitin ligases that target STAT1 and STAT2 for ubiquitination, as well as the mechanisms regulating these events, remain poorly understood. Moreover, the extent of STAT2 stability regulation is not well defined, especially in relation to immune responses and carcinogenesis. Recently, understanding of the role of STAT2 in skin carcinogenesis was profoundly advanced. Specifically, a study found that an environmental stimulus, UVB, modulated STAT2 stability^22^. This is the first result showing that UVB stimulation induces protein–protein interactions between STAT2 and FBXW7, a member of the F-box protein family selectively recognizing target substrates. This mechanistic molecular study was able to demonstrate that GSK3β-induced phosphorylation of the DBD of STAT2, especially at residues Ser381, Thr385, and Ser393, created a recognition degron motif for FBXW7 by forming hydrogen bonds^22^. The physiological relevance of the UVB–STAT2–FBXW7 signaling axis in melanoma formation was investigated, and STAT2 and FBXW7 protein levels showed an inverse correlation in normal and skin cancer tissues (*n* = 77)^22^ (Fig. 4). Since most studies investigating the role of STAT2 have focused only on skin cancer, the various underlying roles of STAT2 in different cellular contexts have been easy to overlook. Moreover, protein–protein interactions induce dramatic conformational changes. Since JAK activation promotes subsequent tyrosine phosphorylation of STAT proteins that form transcriptionally active STAT1 homodimers or STAT1/STAT2/IRF9 complexes^20^, the importance of protein ubiquitination in regulating cytokine signaling and IFN-mediated STAT signaling is underscored by the propensity of tumor cells and viruses to hijack this mode of regulation to evade IFN control and interfere with the ability of a host to suppress malignant growth and viral replication^103,104^.

## Conclusion

The data indicate that STAT2 is a key player in ISG expression in response to IFN stimulation. The molecular mechanisms that modulate ISG expression have been continuously studied by focusing on host defense against foreign invaders such as viruses, and it has been shown that STAT2-associating ISGF3 complexes play essential roles in immune responses, including the activation of immune cells, immune cell propagation, and inflammatory cytokine production. Interestingly, the signaling pathway mediated by STAT2 has also been shown to participate in carcinogenesis, metastasis, and anticancer drug resistance. Moreover, several experimental data have strongly suggested that STAT2 is involved in carcinogenesis, including prostate cancer, renal cancer, leukemia, lymphomas, skin cancer, and melanoma. Regarding molecular mechanisms, although the regulation of STAT2 activity by posttranslational modifications such as phosphorylation is a key event in the formation of the ISGF3 complex and its subcellular localization, a recent publication suggests that STAT2 protein regulation by ubiquitination-mediated proteasomal degradation is another way to elucidate STAT2 involvement in carcinogenesis. Finally, the identification of the extrinsic and intrinsic stimuli that induce STAT2-mediated carcinogenesis is important for understanding STAT2-mediated signaling in carcinogenesis.
